# Patient Outcomes of a Virtual Reality-Based Music Therapy Pilot in Palliative Care

**DOI:** 10.1089/pmr.2024.0022

**Published:** 2024-07-19

**Authors:** Adreanne Brungardt, Angela Wibben, Prajakta Shanbhag, Debra Boeldt, Jeanie Youngwerth, Amanda Tompkins, Abigail J. Rolbiecki, Heather Coats, A. Blythe LaGasse, Jean S. Kutner, Hillary D. Lum

**Affiliations:** ^1^ Division of Geriatric Medicine, University of Colorado School of Medicine, Aurora, Colorado, USA.; ^2^ University of Colorado Hospital Palliative Care Consult Service, University of Colorado Hospital, Aurora, Colorado, USA.; ^3^ National Mental Health Innovation Center, University of Colorado Anschutz Medical Campus, Aurora, Colorado, USA.; ^4^ Division of General Internal Medicine, University of Colorado School of Medicine, Aurora, Colorado, USA.; ^5^ Department of Family Medicine, University of Colorado School of Medicine, Aurora, Colorado, USA.; ^6^ College of Nursing, University of Colorado Anschutz Medical Campus, Aurora, Colorado, USA.; ^7^ School of Music, Theatre and Dance, Colorado State University, Fort Collins, Colorado, USA.

**Keywords:** hospital-based care, music therapy, palliative care, pilot study, virtual reality

## Abstract

**Background:**

Hospitalized patients with palliative care needs often have high levels of physical and psychological symptom distress. Virtual reality (VR) with a music therapy intervention may improve physical and psychological symptoms.

**Objectives:**

To assess symptom distress and quality of life (QOL) among hospitalized palliative care patients who participated in a virtual reality-based music therapy (VR-MT) intervention, and to explore VR-MT from the perspectives of health care professionals involved in their care.

**Design:**

Single-arm pilot study of a two-day VR-MT intervention.

**Setting/Participants:**

Patients seen by an inpatient palliative care consultation service at a U.S. hospital could participate in the VR-MT intervention. Participants created a customized soundtrack with a music therapist and then listened to it while experiencing a 360-degree VR nature-based environment of their choice.

**Measurements:**

Patients completed the Edmonton Symptom Assessment System, revised version (ESAS-r) and McGill Quality of Life, revised version (MQOL-R) before and after VR-MT. Members of the participants’ health care teams were interviewed.

**Results:**

Seventeen patients completed VR-MT (range 20–79 years of age, 59% women). Moderate clinical improvements were observed for total ESAS-r score (Cohen’s *d* effect size, 0.68), physical distress subscale (0.52), and psychological distress subscale (0.60); small improvements were observed in total MQOL-r score (0.26) and the existential subscale (0.27). Health care team members described the value of VR-MT as facilitating meaningful conversations.

**Conclusions:**

This pilot study of VR combined with a music therapy intervention for hospitalized patients with palliative care needs supports opportunities for future study of potential improvements in symptom distress and QOL.

## Introduction

Palliative care is team-based, multidimensional care that utilizes a holistic approach to symptom management for seriously ill patients.^1^ The importance of nonpharmacologic interventions in palliative care is increasingly recognized, with music therapy and virtual reality (VR) representing two holistic treatment modalities employed to comprehensively approach patient symptoms.^2–5^ Defined by the American Music Therapy Association, music therapy is the clinical and evidence-based use of music interventions to accomplish individualized goals within a therapeutic relationship with a credentialed professional, by addressing physical, emotional, cognitive, and social needs.^6^ Music therapy for patients with palliative needs can include a broad range of techniques such as therapeutic singing, music-assisted relaxation, creative self-expression through music, and supportive verbal processing through music.^7–10^ Music therapy addresses core components of palliative care including decreasing pain and improving patient-reported outcomes such as spiritual well-being, depression, and stress.^11–13^

VR is a technology that uses computer-generated simulations or 360-video to create immersive, interactive environments for its users. Delivery of VR typically involves an all-in-one standalone headset to transport the user into an immersive 360-degree virtual environment. Though VR is often used for entertainment, education, and training, there has been over two decades of research citing the benefits of VR on health outcomes.^14^ These studies have taken place in a variety of settings, including ambulatory care, acute or intensive care, and more recently, in palliative care.^14–17^ Existing research emphasizes the therapeutic benefit of VR for management of chronic pain,^18^ mental health outcomes (e.g., anxiety and depression),^19^ and symptom management in the supportive and palliative care setting.^20,21^ In the context of palliative care, a scoping review identified 10 studies using VR in a variety of palliative care settings and reported positive data related to usability, feasibility, and acceptability, as well as the need for evidence on biopsychosocial patient outcomes.^17^

The application of technology to music therapy practice has primarily involved electronic media, mobile applications, adaptive instruments, and production software for therapeutic music video production, memory keepsake creation through composition and recordings, and personalized music experiences.^22^ VR provides an opportunity to uniquely link a fully immersive visual sensory experience with a therapeutic music intervention. Despite the growing body of research underscoring the therapeutic benefits of music therapy and immersive VR as standalone interventions, there is limited evidence that has explored the joining of immersive VR and music therapy as a holistic approach to symptom management in the palliative care setting. If such synergistic interventions are feasible and improve patient outcomes, it will also be important to study the biopsychosocial mechanisms involved in such VR-mediated visual sensory experiences that augment evidence-based music therapy approaches.

We created and demonstrated the feasibility, acceptability, and usability of a novel VR-based music therapy (VR-MT) intervention for hospitalized palliative care patients consisting of creating a personalized soundtrack with a music therapist who is part of the palliative care team, and then pairing listening to the soundtrack with a 360-degree immersive, nature-based VR environment.^23^ The objective of this article is to explore (1) potential improvements in symptom distress and quality of life (QOL) among hospitalized palliative care patients who participated in the VR-MT intervention, (2) rating of VR-MT by patients, and (3) health care professional perspectives on the value of VR-MT for their patients.

## Materials and Methods

### Design

This six-month single-arm pilot study describes outcomes of hospitalized palliative care patients who participated in a VR-MT intervention, using quantitative patient-reported outcome measures and brief qualitative interviews with health care team members to understand the potential value of integrating the VR-MT experience into hospital-based palliative care. The pilot was conducted over a relatively short six-month period to determine feasibility of collecting patient-reported outcomes; it was not powered to detect efficacy. This pilot study was approved by the Colorado Multiple Institutional Review Board (Protocol #19-1672).

### Clinical palliative care program setting

The study was conducted at University of Colorado Hospital, an academic teaching hospital in Aurora, Colorado. The Palliative Care Service, established in 1998, provides hospital-based consultative and outpatient clinic-based care. The palliative care service is staffed by four interprofessional teams of specialty palliative-trained board-certified physicians, advanced practice practitioners, social workers, chaplains, palliative care volunteers, a nurse care manager, a board-certified music therapist, and a board-certified art therapist. The inpatient consultative service is available seven days a week, including on-call coverage. Inpatient referrals are initiated by the primary hospital teams. In fiscal year 2020, 1250 new inpatient consultations were conducted with an average hospital length of stay of 12.3 days.

### Participants

Potentially eligible patients for VR-MT were identified from the inpatient palliative care service during daily interprofessional palliative care team rounds. Adult (age 18+) patients with a diagnosis of cancer, heart failure, or end-stage renal disease who had a palliative care consultation and estimated length of stay of at least two days were eligible. These diagnoses were selected for this feasibility pilot based on input from the palliative care team that patients with these diagnoses are sufficiently common, readily identifiable, and felt to have relatively low presence of delirium or cognitive impairment. Patients were excluded if they were deaf, legally blind, or unable to provide informed consent for the study. The palliative care team provided patients with an informational flyer. If a potential participant was interested, a research assistant was notified by a palliative care team member, and then the research assistant described the study purpose and research activities to the patient and answered questions. Prior to enrollment, a research assistant administered the Confusion Assessment Method to screen for delirium, recognizing that delirium significantly limits an individual’s ability to participate in the informed consent process and study activities (VR-MT intervention and data collection).^24^ Eligible and interested patients provided written informed consent. Patient recruitment occurred from September 2019 through February 2020 (and thus, was not affected by the COVID-19 pandemic). Health care professionals who were involved in the care of an enrolled VR-MT participant, such as members of the palliative care team or primary hospital team, were eligible for an interview and approached within one month following the patient’s hospital discharge. Patient participants received $25; health care professionals were not reimbursed.

### Intervention

A detailed VR-MT intervention protocol is published.^23^ *Day 1*: The credentialed music therapist conducted a consultation to identify and explore the patient’s past use and engagement with music, as well as the patient’s hopes and intentions of preferred music listening within a VR environment. The consultation focused on building therapeutic rapport, including establishing a therapeutic space for emotional processing and supportive care in serious illness. This visit resulted in a custom soundtrack, up to 20 minutes, of patient-selected meaningful songs using Apple Music. The music therapist then described the four available 360-degree nature VR videos (Atmosphaeres; Germany) downloaded onto an Oculus Go VR Headset (Facebook; Menlo Park, CA), and noted the participant’s choice of VR video. *Day 2*: The music therapist assisted the patient with adjusting and using the Oculus Go headset and initiated the VR-MT intervention using the patient’s soundtrack and selected VR video. Song selections were streamed through an iPad with external headphones worn over the VR headset to overlay the nature sounds. After the VR experience, the music therapist used a therapeutic debriefing tool (consistent with other music therapy interventions, the tool is standardized, creates space for awareness of physical and emotional responses, discusses opportunities for personal goals after hospitalization, asks about any other needs, and provides closure for the VR-MT intervention) to assist the patient with processing the therapeutic music experience including emotions, reflections, or physiological responses that occurred. Hospital infection control cleaning procedures were followed, including use of antibacterial wipe and ultraviolet sanitation drawers for all materials.

### Data collection

Immediately before and after the Day 2 VR-MT experience, the research assistant administered the survey instruments to assess symptom distress and QOL of the participating patients. To measure symptoms, the 10-item Edmonton Symptom Assessment Scale revised edition (ESAS-r) was used.^25,26^ Patients rated the severity of the 10 symptoms on a 0–10 scale (10 = worse). ESAS subscale scores included total score, physical distress, and psychological distress. Physical distress is the sum of pain, fatigue, nausea, drowsiness, appetite, and shortness of breath. Psychological distress is the sum of anxiety and depression. To measure QOL, the 15-item McGill Quality of Life questionnaire revised edition (MQOL-r) was used.^27,28^ Patients rated each item on a 0–10 scale (10 = worse). MQOL-r subscale scores included total score, physical, psychological, existential, and social subscales. After the intervention, patients also answered three adapted questions from the Mobile App Rating Scale^29^ asking if VR-MT (1) increased awareness of importance of addressing wellbeing, (2) motivation to promote wellbeing, and (3) improved QOL on a scale of 1–5 (questions available upon request).

To explore the value of VR-MT as part of hospital-based palliative care, research team members (A.B. and A.T.) conducted semistructured interviews with health care professionals who were purposively sampled based on their involvement in caring for an enrolled patient. Interviews were conducted in person at the hospital, audio-recorded, and professionally transcribed. Health care professionals were instructed to think about one specific patient at a time and to describe the impact of VR-MT on the patient. They were asked whether having the patient participating in VR-MT influenced the workflow of the clinical team or personal rapport including questions such as “Did you speak with the patient about their experience?”, “Did their participation change your interactions with the patient?” The interview guide is available on request. The music therapist’s clinical notes from the VR-MT intervention were also reviewed to provide context about the patient and intervention.

### Data analysis

We used descriptive statistics to summarize quantitative data using SAS, version 9.4. For both the ESAS-r and MQOL-r, we compared subscales using paired *t*-tests and calculated Cohen’s *d* effect sizes.^30^ As a measure of clinically meaningful changes, a Cohen’s *d* of 0.2 or higher was considered a small effect size; a Cohen’s *d* of 0.5 or higher was considered a moderate effect size. We use a qualitative descriptive approach to analyze interviews and music therapist clinical notes.^31^ Two research team members (A.B. and A.W.) read all transcripts independently, organized verbatim quotes into emergent themes, and met twice over two months with a third team member (H.D.L.) to discuss findings to reach consensus on final themes. No qualitative analytic software program was used. Salient quotations were selected from interviews to demonstrate themes.

## Results

**FIG. 1.**
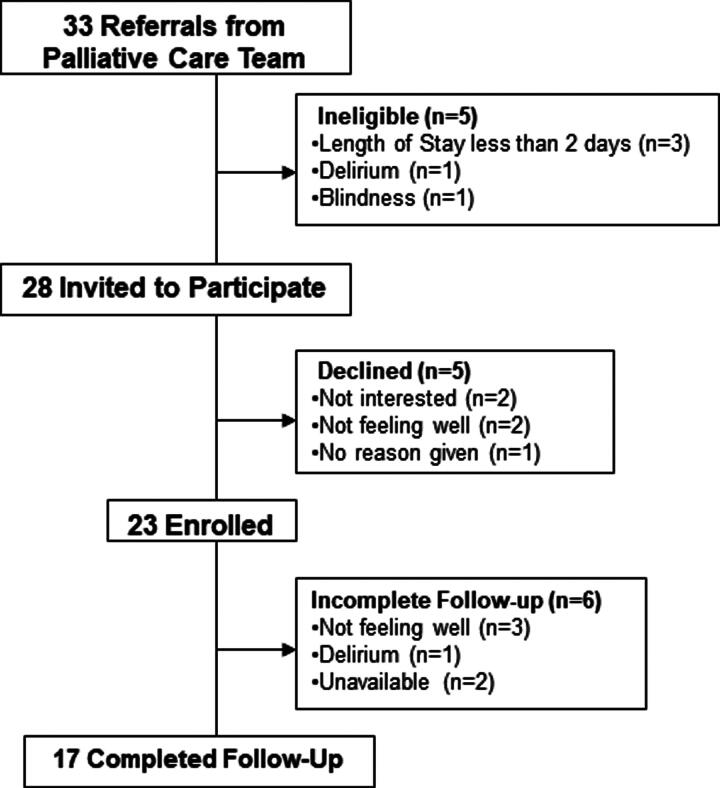
Description of study flow including referrals, eligibility, enrollment, and study completion.^21^

During the enrollment period, 33 patients with palliative care needs were offered the VR-MT intervention; 23 consented and 5 declined (Fig. 1). The most common reasons for declining were “not interested” or “not feeling well.” Six participants enrolled but were then unavailable for intervention completion and follow-up owing to hospital discharge, procedures, or withdrawal from the study owing to not feeling well. Of those who consented, 17 participants completed all VR-MT study components (74% completion). Participants had a mean age of 47 years old; 59% identified as women, 18% identified as Hispanic, and 12% as Black or African American (see Table 1 for all demographic characteristics).

**Table 1.** Characteristics of Patients Who Completed the Virtual Reality-Music Therapy Intervention (*N* = 17)

Characteristic	*N* (%)
Age, years (SD)	47.4 (17.9)
Female gender	10 (59)
Ethnicity	
Hispanic	3 (18)
Non-Hispanic	14 (82)
Race	
White	12 (70)
Black or African American	2 (12)
Asian	1 (6)
Other	2 (12)
Primary diagnosis	
Cancer	9 (53)
Heart failure	6 (35)
End-stage renal	2 (12)

SD, standard deviation.

As shown in Table 2, participants’ report of symptom distress decreased after participating in the VR-MT intervention. Comparison of preintervention versus post-intervention ratings for total ESAS-r score (32.8 vs. 22.5, *p* = 0.007), physical distress subscale (20.9 vs. 15.5, *p* = 0.022), and psychological distress subscale (6.5 vs. 3.8, *p* = 0.036) all improved, with corresponding moderate effect sizes. With respect to QOL, the total MQOL-r score (5.9 vs. 6.8, *p* = 0.003) and three of the four subscales showed improvement after the VR-MT intervention (Table 2). The total MQOL-r score and the existential subscale had small effect sizes with a Cohen’s *d* higher than 0.2. The individual symptoms for the ESAS-r and the MQOL-r are shown in Supplemental Supplementary Table S1.

**Table 2.** Comparison of Palliative Care Patient-Report Outcomes Before and After the Virtual Reality-Based Music Therapy Intervention

Outcome measure	Preintervention mean (SD)	Post-intervention mean (SD)	*p* value	Effect size (Cohen’s *d*)
Edmonton Symptom Assessment System revised				
Total score	32.8 (12.8)	22.5 (17.3)	0.007	0.68
Physical distress subscale	20.9 (8.4)	15.5 (12.1)	0.022	0.52
Psychological distress subscale	6.5 (4.8)	3.8 (4.6)	0.036	0.60
McGill Quality of Life revised				
Total score	5.9 (1.5)	6.8 (1.1)	0.003	0.26
Physical subscale	3.5 (1.9)	4.9 (1.8)	0.002	0.19
Psychological subscale	6.0 (2.3)	6.8 (2.1)	0.18	0.08
Existential subscale	6.2 (1.3)	7.2 (1.2)	0.002	0.27
Social subscale	7.8 (2.2)	8.4 (1.5)	0.047	0.08

Building on patient perspectives on feasibility, usability, acceptability, and user experience of VR-MT as previously reported,^21^ 59% of the participants considered the intervention likely to increase their awareness of the importance of addressing their own wellbeing (score of 4 or 5 on the Mobile App Rating Scale), 65% considered that it was likely to increase their motivation to promote their wellbeing, and 71% considered it likely to increase their QOL.

Most health care professionals (75%) were part of the palliative care team, while others were nurses or rehabilitation therapists on the patient’s primary hospital team. Salient quotations from the health care professionals who participated in interviews are shown in Table 3 to demonstrate the four themes. The four themes were: (1) VR-MT opens doors to meaningful conversations among clinical staff with the patient, (2) acceptable integration of identifying patients for VR-MT into daily palliative care rounds, (3) noticeable changes in the patient after VR-MT, and (4) potential value of ongoing patient use and benefit of intervention components. As an example of how the VR-MT intervention supported meaningful conversations, a palliative care social worker said that reading the music therapist’s documentation from VR-MT “gives me something to talk about to break the ice, to create some sort of a rapport, but always to deepen the understanding of the patient as a person.” Another staff member summarized her impression as “Something was different. Something shifted, something big.” Health care staff’s comments focused more on the music portion of the intervention than the VR component.

**Table 3.** Staff Members’ Perspectives on Virtual Reality-Based Music Therapy Intervention as Part of Inpatient Care

Themes	Exemplar verbatim quotes
Intervention opens doors to meaningful conversations	I was surprised to read that she chose gospel music, and I don’t know a lot about gospel music but it did allow me to see how much spirituality is important in her life…. It gives me something else that maybe I can work with when I work with her, like maybe talking more about that and how could she use her spirituality to help her try to stay on track with (medical intervention). (outpatient care manager)
	We talked about that song because it’s something we both liked…I think that process was really meaningful… it just kind of gave him a pathway into doing some of his end-of-life work and thinking about his legacy. (palliative care chaplain)
Post-intervention patient changes	His affect was not as flat…and there was definitely a decrease in use of pain medicine for a couple of days. (palliative care clinician)
	She was much less, much less labile. She was more grounded…more able to have a coherent conversation. (palliative care chaplain)
	She seemed more peppy. (floor nurse)
Acceptable integration into morning rounds	We kind of have a new format of presenting patients to make sure we’re not talking the whole time about the medical stuff but incorporating the interdisciplinary team input. I think by doing that it brings to mind who might be a VR subject. I don’t think it gets in the way. (palliative care clinician)
	On our morning rounds, we have a nice structure where someone might mention virtual reality… I don’t think it’s a big burden. (palliative care clinician)
Extended patient use and benefits of intervention components	They made the decision to play songs from his playlist during the extubation. (palliative care chaplain)
	She ordered her own VR headset while inpatient (palliative care social worker)
	And the wife actually told us that she thought it was great because he was able to engage in conversations with other people about different things. Like not in the hospital but like friends and family. He had something to talk to them about when he called them on the phone. (palliative care clinician)

VR, virtual reality.

## Discussion

The results of this VR-MT pilot study for hospitalized patients with palliative care needs are promising, suggesting potential for this innovative approach to managing symptoms and improving QOL for patients with serious illness. The observed improvements in the overall ESAS and McGill QOL scales, as well as individual symptoms such as tiredness and anxiety (Supplementary Table S1), underscore the potential tangible impact of combining VR with music therapy as a palliative intervention. Although the changes in the McGill QOL psychological subscale did not reach statistical significance, patients reported improvements in their depression on the ESAS while engaging in VR-MT, which warrants further exploration with a larger sample size, should this be the primary outcome of interest in future clinical studies of VR-MT.

This small study demonstrates the feasibility of measuring patient-reported outcomes, provides initial estimates of clinical effect sizes, and explores descriptions of VR-MT from the perspectives of health care team members involved in the care of these patients. These are each important considerations for planning future trials and real-world implementation. For example, health care staff interviews highlighted the impact of VR-MT on promoting meaningful conversations as part of rapport building. This study was conducted at a single site and with a relatively small number of nonpalliative care health care team members (i.e., hospital floor nurse or rehabilitation therapist); further study is needed to understand barriers and facilitators to implementation in the context of hospitalization. It should be noted that in this single-site study, the specialty palliative care hospital staff members were likely to have more awareness of music therapy because the study music therapist was well-integrated into the palliative care team.

The application of VR-MT differs from traditional music therapy intervention because traditional music therapy has more flexibility than the VR-MT intervention protocol. In traditional music therapy, interventions are chosen and the music is selected based on the individual patient/client’s assessment, treatment plan, and/or therapeutic goals within the session or therapy trajectory. Future studies are needed to investigate this protocolized delivery of VR-MT compared with music therapy consultation and preferred music listening that does not include VR. Further investigation is also needed to explore mechanisms or factors of VR-MT that may contribute to improvement in outcomes like anxiety, tiredness, wellbeing, and depression, and also to provide insight to the intervention’s overall effectiveness in terms of managing symptoms and improving QOL. Longer follow-up might also hint at the sustainability of observed benefits, and help tailor the intervention for patients from diverse cultural backgrounds, or for patients in other supportive care settings (e.g., those who are not at end-of-life but utilizing palliative support in their care setting). Additionally, as VR technology changes, there is a lot of potential for personalization and mood tracking within the VR environment. Future studies should explore a larger library of immersive content to increase personalization.

This study has several limitations. Patients were not randomized and there was no control arm for comparison. The study included a small convenience sample of patients and was not powered to test efficacy. Specific to the intervention, there were a smaller number of nature-based VR environments available on the VR headset, compared with the large number of music selections for the personalized VR-MT intervention. A prior evaluation of participants’ feedback noted a desire for more video choices and interaction from the VR environment, including in relationship to the personalized soundtrack.^23^ Nonetheless, given the measurable improvements in this small sample, VR-MT (or current state-of-the-art interventions that combine VR technology with music therapy approaches) warrants feasibility testing in a randomized controlled trial that has a larger and more representative sample and can provide preliminary efficacy estimates for comparing VR-MT with a control condition.

## Conclusions

Results of this pilot study support further research to investigate VR-MT as means for improvement in symptom distress and QOL for hospitalized palliative care patients. While more studies are being published on use of VR in various health care settings including palliative care, this intervention is the first to combine VR with music therapy. This promising intersection of VR technology joined with music therapy provides avenues for holistic approaches for managing physical, psychological, existential, and social distress and improving QOL for hospitalized patients with palliative care needs. Continued research will help refine and validate the VR-MT intervention’s efficacy and optimize integration in palliative care clinical workflows.

## Abbreviations Used

ESAS-r: Edmonton Symptom Assessment Scale revised edition
MQOL-r: McGill Quality of Life revised edition
QOL: quality of life
SD: standard deviation
VR: Virtual reality
VR-MT: Virtual reality-based music therapy

## References

[1] Ferrell BR, Twaddle ML, Melnick A, et al. National consensus project clinical practice guidelines for quality palliative care guidelines, 4th Edition. J Palliat Med 2018;21(12):1684–1689; doi: 10.1089/jpm.2018.0431 30179523

[2] Huda N, Banda KJ, Liu AI, et al. Effects of music therapy on spiritual well-being among patients with advanced cancer in palliative care: A meta-analysis of randomized controlled trials. Semin Oncol Nurs 2023;39(6):151481; doi: 10.1016/j.soncn.2023.151481 37541810

[3] Huang E, Huang J. Music therapy: A noninvasive treatment to reduce anxiety and pain of colorectal cancer patients-a systemic literature review. Medicina (Kaunas) 2023;59(3); doi: 10.3390/medicina59030482 PMC10051791 36984483

[4] Bruno RR, Wolff G, Wernly B, et al. Virtual and augmented reality in critical care medicine: The patient’s, clinician’s, and researcher’s perspective. Crit Care 2022;26(1):326; doi: 10.1186/s13054-022-04202-x 36284350 PMC9593998

[5] Rusowicz J, Szczepańska-Gieracha J, Kiper P. Neurologic music therapy in geriatric rehabilitation: A systematic review. Healthcare (Basel) 2022;10(11); doi: 10.3390/healthcare10112187 PMC9690210 36360527

[6] What is Music Therapy? | What is Music Therapy? | American Music Therapy Association (AMTA). [Accessed [Last accessed: April 7, 2024]. Available from: https://www.musictherapy.org/

[7] Srolovitz M, Borgwardt J, Burkart M, et al. Top ten tips palliative care clinicians should know about music therapy and art therapy. J Palliat Med 2022;25(1):135–144; doi: 10.1089/jpm.2021.0481 Epub 2021 Oct 19. 34665661

[8] Perez-Eizaguirre M, Vergara-Moragues E. Music therapy interventions in palliative care: A systematic review. J Palliat Care 2021;36(3):194–205; doi: 10.1177/0825859720957803 32928042

[9] Clements-Cortes A. Development and efficacy of music therapy techniques within palliative care. Complement Ther Clin Pract May 2016;23:125–129; doi: 10.1016/j.ctcp.2015.04.004 25986297

[10] Wood C, Cutshall SM, Wiste RM, et al. Implementing a palliative medicine music therapy program: A quality improvement project. Am J Hosp Palliat Care 2019;36(7):603–607; doi: 10.1177/1049909119834878 30845807

[11] Gutgsell KJ, Schluchter M, Margevicius S, et al. Music therapy reduces pain in palliative care patients: A randomized controlled trial. J Pain Symptom Manage 2013;45(5):822–831; doi: 10.1016/j.jpainsymman.2012.05.008 23017609

[12] Warth M, Koehler F, Brehmen M, et al. “Song of Life”: Results of a multicenter randomized trial on the effects of biographical music therapy in palliative care. Palliat Med 2021;35(6):1126–1136; doi: 10.1177/02692163211010394 33876660 PMC8188998

[13] Archie P, Bruera E, Cohen L. Music-based interventions in palliative cancer care: A review of quantitative studies and neurobiological literature. Support Care Cancer 2013;21(9):2609–2624; doi: 10.1007/s00520-013-1841-4 23715815 PMC3728458

[14] Garrett BM, Tao G, Taverner T, et al. Patients perceptions of virtual reality therapy in the management of chronic cancer pain. Heliyon May 2020;6(5):e03916; doi: 10.1016/j.heliyon.2020.e03916 32426540 PMC7226660

[15] Son H, Ross A, Mendoza-Tirado E, et al. Virtual reality in clinical practice and research: Viewpoint on novel applications for nursing. JMIR Nurs 2022;5(1):e34036; doi: 10.2196/34036 35293870 PMC8968556

[16] Bruno RR, Bruining N, Jung C. Virtual reality in intensive care. Intensive Care Med 2022;48(9):1227–1229; doi: 10.1007/s00134-022-06792-0 35816236 PMC9272874

[17] Moloney M, Doody O, O’Reilly M, et al. Virtual reality use and patient outcomes in palliative care: A scoping review. Digit Health 2023;9:20552076231207574; doi: 10.1177/20552076231207574 37928326 PMC10621306

[18] Guo Q, Zhang L, Gui C, et al. Virtual reality intervention for patients with neck pain: Systematic review and meta-analysis of randomized controlled trials. J Med Internet Res 2023;25:e38256; doi: 10.2196/38256 37010891 PMC10131665

[19] Wiebe A, Kannen K, Selaskowski B, et al. Virtual reality in the diagnostic and therapy for mental disorders: A systematic review. Clin Psychol Rev 2022;98:102213; doi: 10.1016/j.cpr.2022.102213 36356351

[20] Rolbiecki AJ, Craig K, Megan P, et al. Virtual reality and neurofeedback for management of cancer symptoms: A feasibility pilot. Am J Hosp Palliat Care 2023;40(3):291–298; doi: 10.1177/10499091221109900 35723043

[21] Rolbiecki AJ, Govindarajan A, Froeliger B. Immersive virtual reality and neurofeedback for the management of cancer symptoms during treatment. Support Care Cancer 2023;31(8):493; doi: 10.1007/s00520-023-07957-3 37493785

[22] Crowe BJ, Rio R. Implications of technology in music therapy practice and research for music therapy education: A review of literature. J Music Ther 2004;41(4):282–320. 15762835 10.1093/jmt/41.4.282

[23] Brungardt A, Wibben A, Tompkins AF, et al. Virtual reality-based music therapy in palliative care: A pilot implementation trial. J Palliat Med May 2021;24(5):736–742; doi: 10.1089/jpm.2020.0403 33227225 PMC8064967

[24] Inouye SK, van Dyck CH, Alessi CA, et al. Clarifying confusion: The confusion assessment method. A new method for detection of delirium. Ann Intern Med 1990;113(12):941–948. 2240918 10.7326/0003-4819-113-12-941

[25] Bruera E, Kuehn N, Miller MJ, et al. The Edmonton Symptom Assessment System (ESAS): A simple method for the assessment of palliative care patients. J Palliat Care 1991;7(2):6–9. 1714502

[26] Lopez G, Christie AJ, Powers-James C, et al. The effects of inpatient music therapy on self-reported symptoms at an academic cancer center: A preliminary report. Support Care Cancer 2019;27(11):4207–4212; doi: 10.1007/s00520-019-04713-4 30825024

[27] Cohen SR, Mount BM, Bruera E, et al. Validity of the McGill Quality of Life Questionnaire in the palliative care setting: A multi-centre Canadian study demonstrating the importance of the existential domain. Palliat Med 1997;11(1):3–20; doi: 10.1177/026921639701100102 9068681

[28] Cohen SR, Sawatzky R, Russell LB, et al. Measuring the quality of life of people at the end of life: The McGill Quality of Life Questionnaire-Revised. Palliat Med 2017;31(2):120–129; doi: 10.1177/0269216316659603 27412257

[29] Stoyanov SR, Hides L, Kavanagh DJ, et al. Mobile app rating scale: a new tool for assessing the quality of health mobile apps. JMIR Mhealth Uhealth 2015;3(1):e27; doi: 10.2196/mhealth.3422 25760773 PMC4376132

[30] Cohen J. (1988). Statistical power analysis for the behavioural sciences (2nd ed.). Lawrence Erlbaum Associate Publishers; London.

[31] Sandelowski M. Whatever happened to qualitative description? Res Nurs Health 2000;23(4):334–340. 10940958 10.1002/1098-240x(200008)23:4<334::aid-nur9>3.0.co;2-g

