# Supplementary material for: Patient Outcomes of a Virtual Reality-Based Music Therapy Pilot in Palliative Care
Source: Palliat Med Rep. 2024 Jul 19;5(1):278–85. doi: 10.1089/pmr.2024.0022 (PMC11271146; doi:10.1089/pmr.2024.0022)
Supplement: Supplementary Table S1 [file pmr.2024.0022_supplementaltable1.docx]

Supplemental Table 1. Comparison of Individual Symptoms and Quality of Life Items before and after VR-MT Intervention

|  | **Pre-intervention**  **Mean (SD)** | **Post-intervention**  **Mean (SD)** | **p-value** |
| --- | --- | --- | --- |
| **Edmonton Symptom Assessment System Revised, Individual Symptom** |  |  |  |
| Pain | 3.47 (2.5) | 2.59 (2.3) | 0.29 |
| Tiredness | 5.65 (2.1) | 3.18 (2.6) | **0.005** |
| Drowsiness | 3.23 (2.9) | 2.53 (2.6) | 0.46 |
| Nausea | 1.18 (2.0) | 1.12 (2.6) | 0.94 |
| Lack of Appetite | 4.82 (3.6) | 4.06 (3.6) | 0.54 |
| Shortness of Breath | 2.59 (2.1) | 2.06 (2.6) | 0.51 |
| Depression | 2.59 (2.8) | 1.53 (2.4) | 0.25 |
| Anxiety | 3.94 (2.6) | 2.24 (2.3) | 0.054 |
| Wellbeing | 5.0 (2.3) | 3.06 (2.9) | **0.042** |
| **McGill Quality of Life Revised – Individual Items** |  |  |  |
| Considering all parts of my life (for example, physical, emotional, social, spiritual, and financial) over the past two days (48 hours) the quality of my life was: (0) very bad - (10) excellent | 5.71 (2.1) | 6.41 (1.9) | 0.31 |
| Over the past two days (48 hours) my physical symptoms (such as pain, nausea, tiredness, and others) were: (0) not a problem - (10) a tremendous problem | 6.65 (2.4) | 4.47 (3.0) | **0.027** |
| Over the past two days (48 hours) I felt: (0) physically terrible - (10) physically well | 4.65 (2.6) | 5.82 (1.63) | 0.12 |
| Over the past two days (48 hours), being physically unable to do the things I wanted was: (0) not a problem - (10) a tremendous problem | 7.47 (1.9) | 6.59 (2.4) | 0.25 |
| Over the past two days (48 hours), I was depressed: (0) not at all - (10) extremely | 3.94 (2.8) | 2.76 (2.7) | 0.22 |
| Over the past two days (48 hours), I was nervous or worried: (0) not at all - (10) extremely | 3.94 (2.9) | 3.35 (2.3) | 0.53 |
| Over the past two days (48 hours), I felt sad: (0) never - (10) always | 4.35 (3.0) | 3.53 (2.7) | 0.41 |
| Over the past two days (48 hours), when I thought of the future, I was: (0) not afraid - (10) terrified | 3.65 (2.9) | 3.0 (2.8) | 0.51 |
| Over the past two days (48 hours), my life was: (0) utterly meaningless and without purpose - (10) very purposeful and meaningful | 7.23 (2.2) | 8.0 (2.1) | 0.31 |
| When I think about my whole life, I feel that in achieving life goals I have: (0) made no progress whatsoever - (10) progressed to complete fulfillment | 6.76 (1.8) | 7.35 (1.7) | 0.35 |
| Over the past two days (48 hours), I felt that the amount of control I had over my life was: (0) not a problem - (10) a tremendous problem | 6.12 (2.7) | 4.94 (2.6) | 0.21 |
| Over the past two days (48 hours), I felt good about myself as a person. (0) completely disagree - (10) completely agree | 6.94 (2.05) | 8.18 (1.5) | 0.051 |
| Over the past two days (48 hours) communication with the people I care about was: (0) difficult - (10) very easy | 7.29 (2.9) | 7.59 (2.5) | 0.75 |
| Over the past two days (48 hours) I felt my relationships with the people I care about were: (0) more distant than I would like - (10) very close | 7.29 (3.2) | 8.29 (1.9) | 0.28 |
| Over the past two days (48 hours), I felt supported: (0) not at all - (10) completely | 8.82 (1.6) | 9.41 (1.1) | 0.21 |
